# Evaluation of texture features at staging liver fibrosis based on phase contrast X-ray imaging

**DOI:** 10.1186/s12938-018-0612-3

**Published:** 2018-12-03

**Authors:** Jing Wang, Ming Wang, Song Gao, Hui Li

**Affiliations:** Department of Medical Physics, School of Foundational Education, Peking University Health Science Center, Beijing, 100191 China

**Keywords:** Liver fibrosis, Mouse liver specimen, Phase contrast imaging, Texture features, Neural network

## Abstract

**Background:**

The purpose of this study is to explore the potential of phase contrast imaging to detect fibrotic progress in its early stage; to investigate the feasibility of texture features for quantified diagnosis of liver fibrosis; and to evaluate the performance of back propagation (BP) neural net classifier for characterization and classification of liver fibrosis.

**Methods:**

Fibrous mouse liver samples were imaged by X-ray phase contrast imaging, nine texture measures based on gray-level co-occurrence matrix were calculated and the feasibility of texture features in the characterization and discrimination of liver fibrosis at early stages was investigated. Furthermore, 36 or 18 features were applied to the input of BP classifier; the classification performance was evaluated using receiver operating characteristic curve.

**Results:**

The phase contrast images displayed a vary degree of texture pattern from normal to severe fibrosis stages. The BP classifier could distinguish liver fibrosis among normal, mild, moderate and severe stages; the average accuracy was 95.1% for 36 features, and 91.1% for 18 features.

**Conclusion:**

The study shows that early stages of liver fibrosis can be discriminated by the morphological features on the phase contrast images. BP network model based on combination of texture features is demonstrated effective for staging liver fibrosis.

## Background

Liver fibrosis refers to the abnormal proliferation of connective tissues, such as collagen fiber, elastic fiber, and matrix components amino polysaccharide hyperplasia. It is more prominent in the case of collagen fiber. The disease is potentially caused by various types of chronic liver lesions, such as alcohol, viruses, and drugs. It is a long process of dynamic development. During its initial stage, Liver fibrosis can regress if the cause is reversible. Therefore, it is of great importance from the clinical point of view to detect and assess the fibrosis at early stage, as well as to monitor the liver fibrogenic progress and to guide the therapeutic intervention.

To date, liver biopsy is still considered as the reference test to assess the severity of liver fibrosis. Samples are paraffin-embedded, and cut for several micrometers. The slices are then stained, dehydrated and imaged by optical microscopic. On the basis of the grading standard, the pathologists assess the fibrosis severity according to their experiences. Since the formation of liver fibrosis is a long and complicated process, there may be significant difference in a local region, and also, the process of fibrosis with static and active shows discontinuity. Thus, liver biopsy examinations may lead to false negative results due to inadequate liver tissue sampling. The research by Bedossa et al. shows that even if the length of liver biopsy specimens reaches 25 mill, there is still 25% misjudgment rate of fibrosis [1]. Moreover, liver biopsy is an invasive procedure with side effect of pain or even death, hence it is infeasible to repeat the process frequently.

With the development of imaging techniques, imaging modalities such as ultrasonography, computed tomography (CT) and magnetic resonance imaging (MRI) are becoming more effective measures for assessing the fibrous disease [2–5]. Studies have shown that these methods can detect some signs of liver fibrosis, such as the stiffness, outline, and size of the liver. The associated findings relate to blood vessel diameter, blood flow direction, etc. However, these means are proven not to be sensitive enough to diagnose the fibrous disease due to the limited sensitivity and specificity [6].

Texture patterns are important features for diagnosing of the fibrosis on CT or MR images. Accurate assessment of the disease based on the fibrous morphology changes is clinically challenging, especially for inexperienced residents or general radiologists. In recent years, computer-aided diagnosis (CAD) based on texture features were developed to assess the fibrosis. By choosing small sizes of regions of interest (ROI), textures features were quantified and classification of fibrosis was obtained. However, these studies were mainly concerning the cirrhotic and non-cirrhotic stages, it cannot differentiate the fibrosis, especially at the early stages, and also, the accuracy rate is low owing to the limited resolution of the images [7, 8].

X-ray phase contrast imaging modality has gained tremendous attention these years [9–13], it has already been demonstrated to have high contrast and spatial resolution for visualizing biological soft tissues that have similar attenuation characteristics [14–16]. Analyzer-based phase contrast imaging (ABPCI) method utilizes refraction effect of X-rays with sample, thus, it is particularly sensitive to the boundaries between different tissues, resulting in greatly enhanced image contrast. Studies have demonstrated great potential for medical applications. Li et al. have applied the imaging modality to rat liver samples, and found that liver fibrosis could be discriminated for different stages, and the selected features were found to reflect the coarseness of liver textures [17].

This study mainly focused on the investigation of effectiveness of texture features for quantified diagnosis of liver fibrosis; moreover, the performance of neural net classifier for the characterization and classification of fibrosis was evaluated.

## Materials and methods

### Samples

The samples of fibrous mouse liver were prepared in Peking University Health Science Center. Hepatic fibrosis in mouse was induced by carbon tetrachloride (CCL_4_). The liver specimens were fixed in 10% formalin solution. Sections with thickness of approximately 2.0 mm were cut for ABPCI imaging. Procedures involving animals and their care were conducted in conformity with NIH guidelines (NIH Pub. No. 85–23, revised 1996). After imaging, the samples were dehydrated and paraffin embedded, serial sections of 5 μm were cut and stained for Massontrichrome. The histology pictures were reviewed by a pathologist from Peking University Third Hospital. The livers’ fibrosis stages were evaluated according to the METAVIR scoring system [6], which included four stages (F1–F4), from mild to cirrhosis. In this paper, the liver samples of normal (F0), mild (F1), moderate (F2) and severe (F3) fibrosis were included for imaging analysis.

### Phase contrast imaging

The interaction of X-rays with matter can be expressed as $n = 1 - \delta - i\beta$. The real part *δ* represents the phase term, while the imaginary part *β* corresponds to the absorption. Conventional X-ray imaging is a kind of absorption contrast imaging modality; it uses the absorption term as image contrast. Phase-contrast X-ray imaging utilizes phase term as image contrast. For hard X-rays, the phase term can be approximately 1000 times greater than the absorption term; therefore, the phase contrast imaging techniques are more efficient than the conventional methods for imaging soft tissues. As a kind of phase contrast imaging, ABPCI technique is accomplished by inserting an analyzer crystal between the sample and the detector. X-rays passing through borders of tissues will deviate from their original directions due to refract effect, the refraction causes small angular changes in the beam. The analyzer crystal converts the refraction change into intensity change, resulting in enhancement of the image contrast.

### Experiments

The ABPCI images were acquired at the beamline 4W1A of Beijing synchrotron radiation facility (BSRF). The experimental hutch is approximately 43 m downstream from the source, consisting of two Si (111) crystals, one acts as the monochromator crystal and the other one acts as the analyzer crystal, the tunable energy range is 8–22 keV. During the experiment, the X-ray energy was set at 12 keV. A CCD camera with an effective pixel size of 10.9 μm was used to acquire projection images. X-rays are first monochromatic by the Si (111) crystal, the transmitted beams then strike the sample. They are absorbed, scattered, and diffracted by small angles (of the order of a few microradians) due to the tiny variations in the refractive index of tissues. The emerging refracted and scattered X-rays then hit the analyzer crystal. Only those X-rays which satisfy the Bragg condition of the analyzer crystal can be diffracted by the analyzer on to the detector, others are rejected. The angular acceptance is called the rocking curve (RC) of the crystal. Images are obtained by turning the analyzer at different positions of the RC. At the peak position, images acquired similar to conventional absorption but with enhanced contrast due to the scatter rejection. When setting the analyzer on the full width at half maximum (FWHM) positions of the RC, the slop images are acquired, where the refraction effect is heighted, boundaries within tissues are greatly heightened [10, 18].

In this study, the ABPCI images were acquired at the FWHM angular positions along the RC in order to acquire the best contrast images of hepatic tissues. For comparison, conventional absorption contrast images were obtained. To acquire absorption image, samples were set behind the analyzer crystal, just before the CCD camera. The experimental condition of the absorption imaging was the same with that of the phase contrast imaging.

### The texture features

The texture features were quantified by use of gray-level co-occurrence matrix (GLCM), which characterizes statistic levels and spatial relationship of pixels in an image. The commonly used nine texture features, including energy, contrast, homogeneity, correlation, entropy, sum average, sum entropy, difference average, and difference entropy, were evaluated in this study [19].

The FWHM images of normal, mild, moderate and severe fibrosis were selected. Background subtraction was performed first for texture calculation.

### Calculation of texture features

Regions of interest (ROIs) with size of 100*100 pixels were chosen in each image, the step length setting at 20 pixels, within the tissue region. The original images were segmented into 3332 ROIs for the normal, 4695 ROIs for the mild, 4180 ROIs for the moderate, and 4214 ROIs for the severe tissue, respectively. Background regions and large blood vessels within the liver images were excluded.

In this study, two sets of features were used as input to the classifier performance. One was that the proceeding nine texture features were calculated in four regular directions, namely 0°, 45°, 90° and 135°, respectively, and the 36 features were used as input to the classifier. The other was that texture features were calculated in four regular directions first, means on four directions and the corresponding variances were then calculated, thereby 18 features were obtained and used as input to the classifier.

### Back propagation (BP) neural net classifier

BP is based on gradient descent algorithm. It is a kind of classical and successful learning method of feed forward, which is widely used in image classification. In this study, BP classifier was performed in order to evaluate the capability of texture features to distinguish stages of liver fibrosis. In the modelling procedure, 36 or 18 features were applied to the input of the first layer, the signals then propagated through the network, and the output values were read, showing the classified accuracy of fibrosis. The output layer was 4 dimensions (normal, mild, moderate and severe). Neural networks with 10 hidden nodes were chosen for classification.

The data set was randomly divided into a training set of 70% samples, a verification set of 15% samples, and a test set of 15% samples. The sample sizes for training set, test set, and verification set were 11,495, 2463, and 2463, respectively; and a total of 16,421 samples were used.

### Receiver operating characteristic (ROC) curve

Performance of the classifier was evaluated using ROC curve. The ROC curve is a plot of the true positive rate against the false positive rate across all possible choice of cut-points of a diagnostic test. The closer the curve is to the upper left border, the higher the overall accuracy of the test [20].

## Results

### The ABPCI and absorption images

**Fig. 1:**
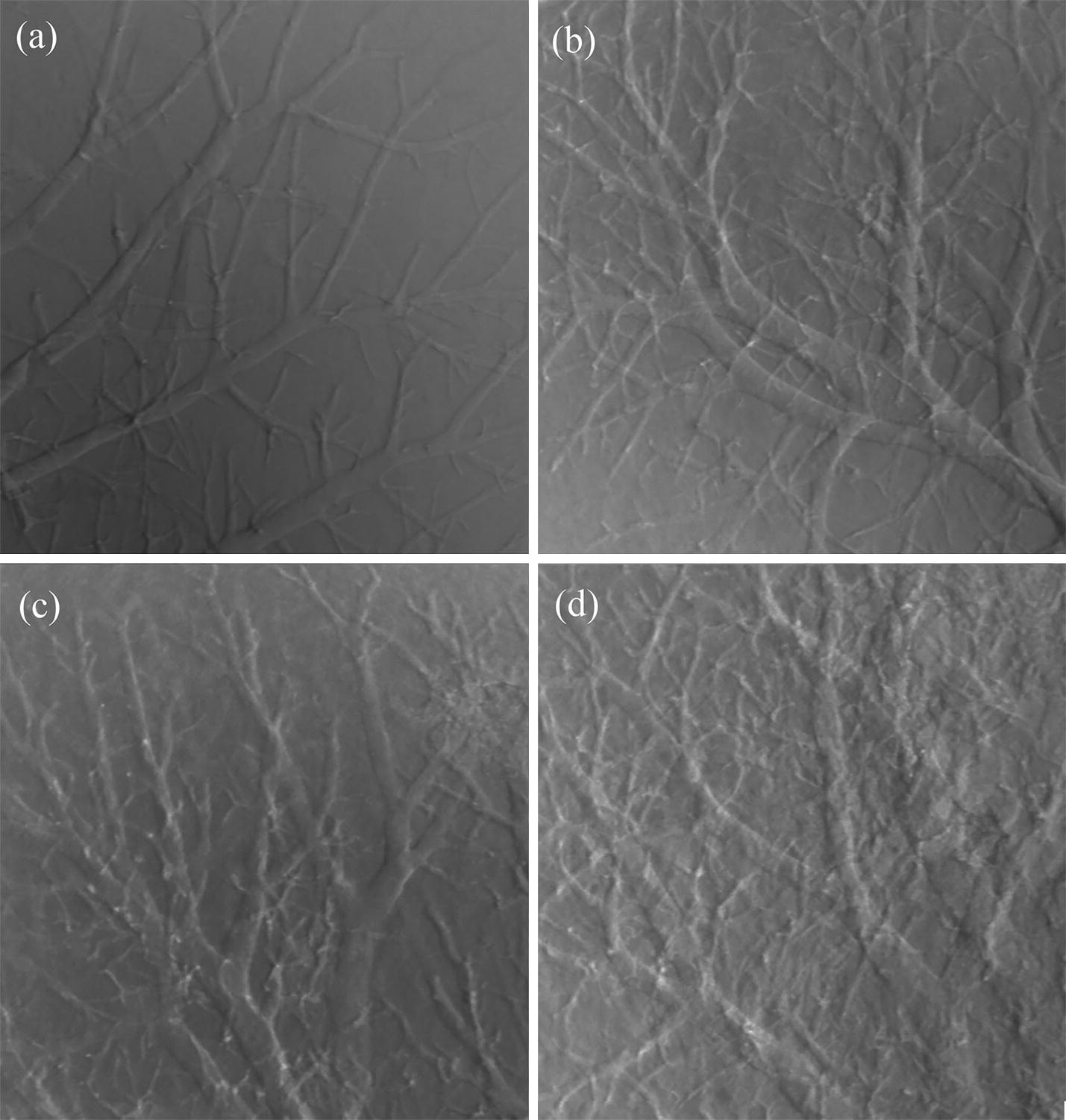
ABPCI images of fibrous samples. **a** Normal. **b** Mild. **c** Moderate. **d** Severe

The ABPCI images of fibrous mouse liver specimen are shown in Fig. 1. The normal liver image (Fig. 1a) shows that liver textures are uniform and homogenous, walls of vessel trees are smooth. Image of mild fibrous liver tissue (Fig. 1b) indicates that liver textures, as well as walls of blood vessels are not smooth anymore. The moderate fibrous image (Fig. 1c) shows that liver textures become rough; blood vessels tend to be distorted. The severe fibrous image (Fig. 1d) reveals fibrous scar tissues; branch vessel structures emerge, indicating blood flow ways reconstruction. The ABPCI images demonstrate vary degrees of texture patterns from normal to severe fibrous stage.

**Fig. 2:**
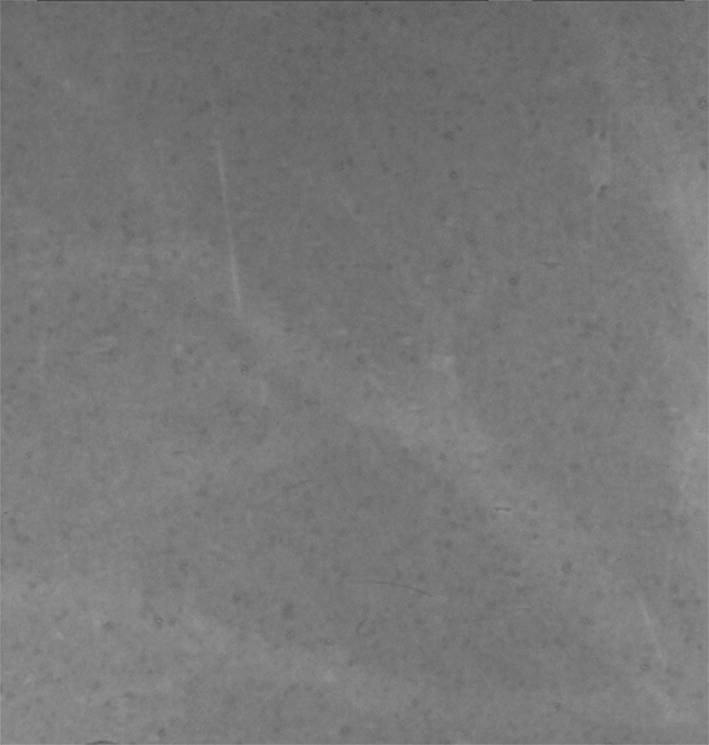
Conventional absorption image of the moderate fibrous sample

Figure 2 shows the conventional absorption contrast image of fibrous liver tissue. Compared to the ABPCI image, liver architecture is almost invisible in the absorption image.

### Calculation of the texture features

#### The probability density functions of 36 features

**Fig. 3:**
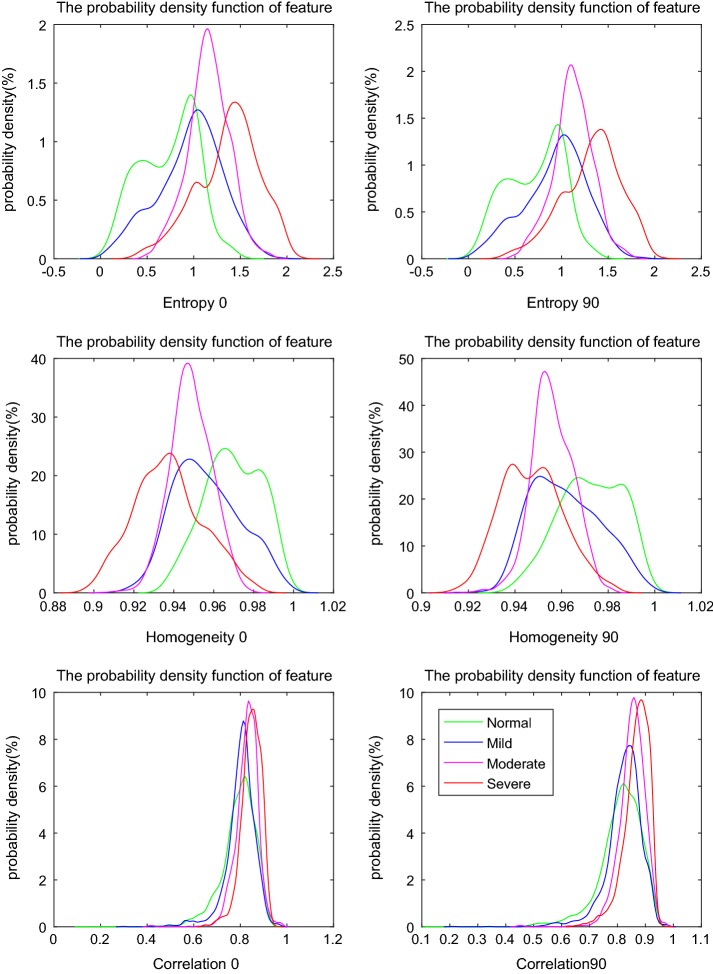
Representative probability density functions of 36 features (on the direction of 0° and 90°)

The above nine texture features based on GLCM in four directions were performed on ABPCI images, and the probability density functions of 36 features were plotted. Through analyzing the curves, we found that there was no significant effect of the direction on the probability density function of feature. Representative results are shown in Fig. 3.

Figure 3 shows that there is no obvious difference in the curves of features on direction of 0° and 90°. For the feature of entropy, there is a clear distinction between normal and severe fibrosis; while for mild and moderate patterns, though they can be discriminated, much overlap exists. Sum entropy is with the same behavior. For the feature of homogeneity, there is a clear distinction between normal and severe fibrosis, while mild and moderate patterns are almost overlapping. Contrast, energy, sum average, difference average and difference entropy are with the same behaviors. The feature of correlation shows almost no difference for degrees of fibrous tissues.

The results also indicate that with the fibrosis progress, the overall values of entropy, contrast, sum average, difference average, sum entropy, and difference entropy tend to increase; while values of homogeneity and energy tend to decrease.

The ability of texture features to differentiate degree of fibrosis is different: entropy and sum entropy show better performances in the classification of fibrosis than features of homogeneity, contrast, energy, sum average, difference average and difference entropy, and the correlation is the least effective. In future studies, it would be more efficient to select features based on these results.

#### The probability density functions of 18 features

**Fig. 4:**
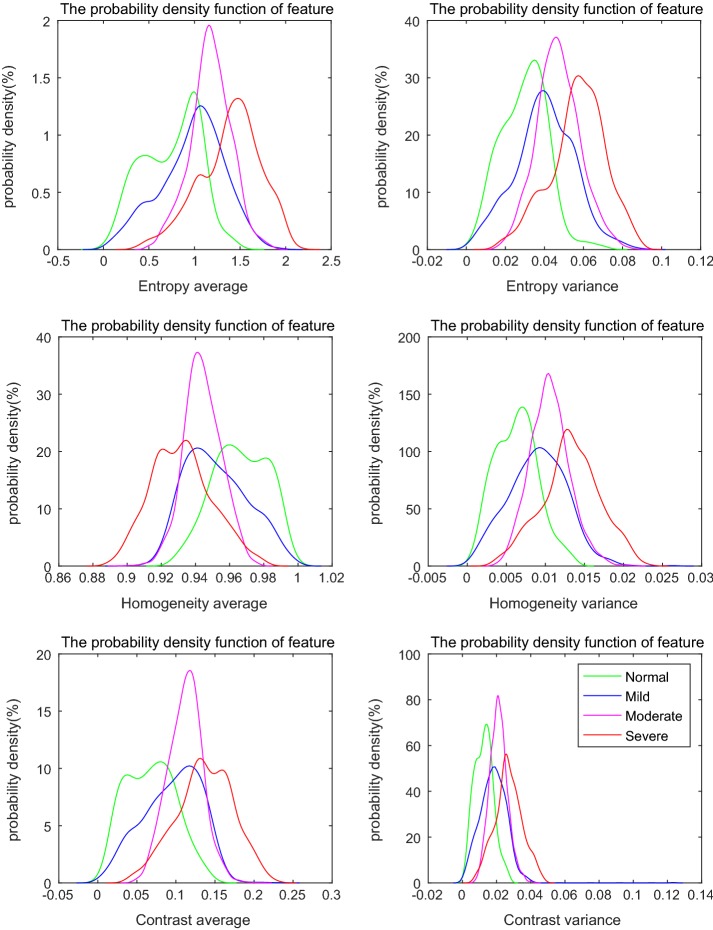
Representative probability density functions of 18 features

The probability density functions of 18 features were obtained. Results of a few representative features are provided in Fig. 4. It shows that characteristics of the averages are similar to those shown in Fig. 3; however, the corresponding variances have special characteristics. The results indicate that no matter how the value of texture feature changes with development of fibrosis, the value of the variance tends to increase; with the normal tissue having the minimal value, and the severe fibrous tissue having the maximal value. The reasons lie in that the normal tissues have smooth surface of liver architectures, with smooth vessel trees. With the development of fibrosis, rough structures emerge; fibrous scar tissues appear, etc., thus, liver architectures become far more volatile. These results indicate that the feature of variance is efficient at reflecting the uniformity of fibrous architectures. Moreover, variances of entropy, sum entropy, and homogeneity possess better discriminative ability for degree of fibrosis than others.

#### Performance of the BP classifier

**Fig. 5:**
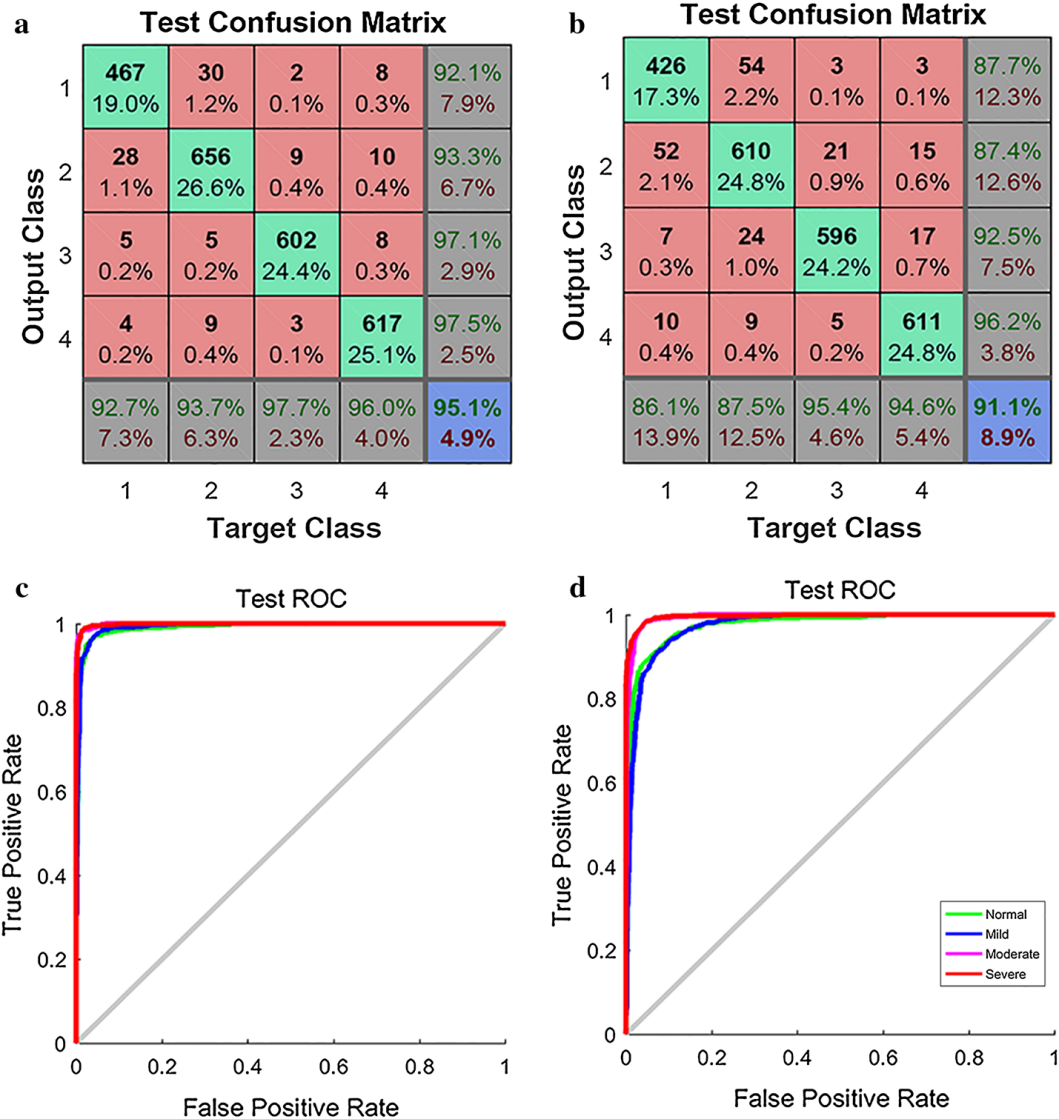
Performance of the BP classifier (1, 2, 3 and 4 represent normal (F0), mild (F1), moderate (F2), and  severe (F3) fibrosis, respectively). **a** Test confusion matrix of BP for 36 features. **b** Test confusion matrix of BP for 18 features. **c** ROC curve for 36 features. **d** ROC curve for 18 features

Confusion matrix of BP neural net classifier was used to evaluate the result of classification. Test confusion matrixes based on 36 and 18 features are shown in Fig. 5a, b. The results of 36 features indicate that 92.7% of normal samples are correctly classified as normal (F0); 93.7% of mild samples are correctly classified as mild (F1); 97.7% of moderate samples are correctly classified as moderate (F2); and 96.0% of severe samples are correctly classified as severe (F3). While for samples which classified by BP as F0, F1, F2, and F3 stages, 92.1%, 93.3%, 97.1%, and 97.5% are classified as F0, F1, F2, and F3 stages by histology. The average accuracy is 95.1%. As for the results of 18 features, the average accuracy is 91.1%, which shows that the classification of 36 features exhibiting better performance than the classification of 18 features, with no significant difference. The result indicates that BP classifier achieves higher recognition rate in moderate and severe fibrosis than in normal and mild fibrosis.

ROC curves based on 36 features and 18 features are shown in Fig. 5c, d. The accuracy rates are higher for the severe and moderate groups than for the normal and mild groups, which is consistent with performance of the confusion matrix. The ROC curve exhibits much high accuracy rate, demonstrating the feasibility and effectiveness of texture features on classification of hepatic fibrosis.

## Discussion

Assessing the severity of fibrosis is really important in therapeutic decision-making. Nowadays, liver biopsy is still used as the reference test to determine the cause and stage of liver fibrosis, and is considered to be the gold-standard. It is an invasive procedure with side effects, including sampling error, subjective interpretation, complicated and costly, etc. In the past decade, noninvasive techniques, such as ultrasonography, CT or MRI have been developed and implanted in clinical practice. Though they are reliable in discriminating early from advanced fibrosis or cirrhosis, they cannot differentiate stages of fibrosis [21].

Texture patterns of hepatic fibrosis are one of the important features to diagnose the liver disease on CT or MRI. In the last decade, computer-aided diagnosis (CAD) based on texture features for CT or MR images were developed to assess the fibrosis. The study by Zhang et al. [7] indicated that MR images had advantages over CT images, with regards to the performance of average accuracy, the best result was about 70% in MR image. The study was mainly concerning the cirrhotic and non-cirrhotic stages; it could not differentiate the fibrosis, especially at the early stage. Besides, the accuracy was low due to the limited resolution of the images.

Phase contrast imaging is specifically sensitive to the refraction properties of tissues; thus, it is able to enhance edges of structures, making soft tissue architectures more discernible in comparison to conventional X-ray imaging. Its potential for medical imaging with micrometer resolution has been explored [15, 16], and the applicability of this imaging modality for visualizing liver fibrosis has been demonstrated previously [17]. This study primarily focuses on the application of the classifier of BP neural network based on textures features obtained from the ABPCI images for characterizing and staging liver fibrosis.

Our study demonstrates image texture calculations based on ABPCI images allow for the quantification of fibrous structures in the early stages. Features of entropy and sum entropy are effective at differentiating fibrosis, possibly due to the fact that these features emphasize edge-related differences between tissues. Although studies based on single feature analysis can be informative, however, the distributions overlap cannot distinguish normal and stages of fibrosis thoroughly; multivariable features are expected to more comprehensively characterize fibrous structures. Therefore, feature combination was utilized as input of the classifier and classification accuracy was evaluated.

BP neural net classifier was exploited as quantification and classification of liver fibrosis, and 36 or 18 features were applied as input of the classifier. Results of the 36 features indicated that no substantial difference in the texture features in four directions, indicating that features in an ROI are approximately rotationally invariant. An average of the feature values over the four directions and the corresponding variances were used as input of the classifier to simplify feature selection and reduce computing time. The higher recognition rate of BP classifier indicates that this method has better applicable effect on staging fibrosis, especially for the early stages. However, selection of combination of texture features for best performance at classifying and staging fibrosis are need to be further studied, and the efficiency and accuracy of the BP classifier in comparison with other classifiers will also need to be investigated. In this regard, those are the focuses of on-going investigations.

Early detection and regular monitoring of fibrosis are challenge for clinical imaging. Our results demonstrated that ABPCI image modality can be a potential imaging method for the early diagnosis of liver fibrosis, and computerized texture features are feasible in classifying the disease.

The results presented in this study were obtained on excised mouse liver specimens. The future research will be extended to in vivo study by using living animals. Moreover, large samples will be utilized to make the study results more efficient and reliable for being viable to clinical practice.

## Conclusions

Mouse fibrous liver samples were imaged by ABPCI. The study demonstrated that early stages of fibrosis can be discriminated by the morphological features of liver tissues. Moreover, texture features were quantified, and BP network model based on combination of features was demonstrated effective for staging fibrosis. These initial experiment results are promising. More research needs to be investigated to make this method a viable imaging and staging technique for liver fibrosis.
